# Segment-Unit Reading Comprehension Training for Japanese Students with Autism Spectrum Disorder and Learning Disabilities

**DOI:** 10.1007/s40617-021-00671-8

**Published:** 2022-02-08

**Authors:** Mikimasa Omori, Junichi Yamamoto

**Affiliations:** 1 Department of Psychology, Graduate School of Human Relations, Keio University, 2-15-45, Mita, Minato-ku, Tokyo 108-8345 Japan; 2 Faculty of Human Sciences, Waseda University, 2-579-15, Mikajima, Tokorozawa-shi, Saitama, 359-1192 Japan; 3 Department of Psychology, Faculty of Letters, Keio University, 2-15-45, Mita, Minato-ku, Tokyo 108-8345 Japan

**Keywords:** Segment-unit reading training, Repeated reading, Reading comprehension, Students with developmental disabilities, Reading difficulties

## Abstract

Students with autism spectrum disorder (ASD) and learning disabilities (LDs) often experience reading difficulties. In particular, reading long passages can cause comprehension problems. We examined whether 8 Japanese students with ASD, 7 students with LDs, and 13 typically developing (TD) students improved their reading comprehension through two types of repeated reading training: whole-sentence-unit reading (WSUR) training and segment-unit reading (SUR) training. Participants undergoing WSUR training read whole sentences repeatedly. In SUR training, they repeatedly read a segment of a sentence in its correct spatial location. Results indicated that students with ASD and LDs showed greater improvement in reading comprehension after SUR training than after WSUR training, whereas both procedures were equally effective for TD students. Moreover, students with ASD showed only negligible reading comprehension improvements, whereas students with LDs showed intermediate improvements after WSUR training. These results suggest that sequentially presenting word segments can improve the reading comprehension of students with ASD and specific LDs.

## Reading Comprehension of Open-Ended Questions

According to the National Institute of Child Health and Human Development (2000), reading comprehension skills comprise “phonemic awareness,” “phonics,” “oral reading fluency,” and “vocabulary” and usually require speaker and listener skills (Greer & Longano, 2010). Reading comprehension requires individuals to make particular responses to particular textual stimuli (Leon et al., 2011). Reading comprehension can be assessed in different ways, with each method offering different purposes. For example, open-ended questions measure different aspects of comprehension from closed-ended questions (Leon et al., 2011). Answering closed-ended questions requires a corresponding stimulus to be selected from multiple stimuli, known as the matching-to-sample (MTS) procedure (Sidman, 1994). In the MTS procedure, individuals match printed texts with a corresponding picture (Sidman, 1994). However, the MTS procedure does not often help develop skills for answering open-ended questions, which require a constructed vocal response and absolute matching to text that involve skills for determining answers to written texts (Goldiamond & Thompson, 2004; Leon et al., 2011). Responses to closed-ended questions are often correlated with prior knowledge, whereas open-ended questions measure the quality of self-explanations (Ozuru et al., 2013). Open-ended questions pose more difficulties for students with autism spectrum disorder (ASD; Nation et al., 2006) and learning disabilities (LDs; Collins et al., 2018), and they need training to improve these skills.

## Presenting Shorter Stimuli During Repeated Reading Training

Fluency is a quick, accurate, and effortless behavioral performance (Johnson & Layng, 1992, 1996). Repeated reading training, in which participants repeatedly read whole sentences until they increase their fluency (Ambruster et al., 2003), is one method of increasing reading fluency. This type of training is referred to as *whole-sentence-unit repeated reading* (WSUR) training in this article. Many studies have indicated that increasing reading fluency could increase comprehension (Fuchs et al., 2001). However, with repeated reading training, some studies have also shown that it was often difficult for individuals to improve their reading comprehension (e.g., Stevens et al., 2017) although they increased reading fluency. Individuals with ASD and LDs often struggle with sentence reading comprehension even though they might be capable of fluent reading and word comprehension (Faggella-Luby & Deshler, 2008; Frith, 1986; Nation et al., 2006). Previous studies have indicated that prereading words from a passage can help students with developmental disabilities read more fluently (Florida Center for Reading Research [FCRR], 2006) and comprehend closed-end questions (Potocki et al., 2015). Therefore, increasing fluent word-reading skills before reading a passage might improve open-ended question comprehension performance.

## Characteristics of Japanese Sentences

However, increasing word-reading fluency may not be sufficient for Japanese students to improve sentence reading comprehension skills because Japanese sentences do not usually have spaces between words. For example, accurately reading the Japanese sentence “かれがりんごをひとつたべた [He ate an apple]” might be easy for Japanese students because the sentence is written in hiragana characters (Japanese phonograms), each part of which controls a part of the spoken response, which has a point-to-point correspondence between letters and sounds (e.g., か is only pronounced /ka/). Japanese first graders can read this sentence as accurately as sixth graders can, even though first graders read less fluently at 202.5 letters per minute compared to sixth graders’ 461.9 letters per minute (Takahashi et al., 2011). Japanese students of different ages might have similar reading accuracy, even though younger students are less fluent than older students because of the nature of the Japanese language.

However, it is often difficult to comprehend the same sentence for Japanese students because they need to divide the sentence into smaller components for comprehension: “かれ (*he* /kare/ [subject]),” “が (/ga/ [subject particle]),” “りんご (*apple* /ringo/ [object]),” “を (/wo/ [object particle]),” “ひとつ (*an* /hitotsu/ [indefinite article]),” “たべた (*ate* /tabeta/ [verb]).” Japanese students with developmental disabilities often struggle with identifying meaningful words in sentences without spaces (Kuhara-Kojima et al., 1996; Takahashi et al., 2011). Therefore, dividing components of sentences might facilitate comprehension for Japanese students with developmental disabilities.

Divided components of Japanese sentences are called segments. Each segment is made of one meaningful word and one meaningless particle (e.g., “かれが [subject and subject particle]”). Hereafter, we will refer to a combination of a word and a particle as *a segment*. Without a particle, who did what for whom cannot be comprehended just by the word order. A word within a single segment has a meaning, whereas a particle has no meaning but refers to the context of the sentence. Therefore, unlike English readers, Japanese students must learn to develop segment-reading fluency skills rather than word-reading fluency.

## Repeated Segment Reading by Japanese Students with Developmental Disabilities

Recent studies have shown that repeatedly reading sentence segments facilitates Japanese reading comprehension skills for closed-ended (Omori & Yamamoto, 2018) and open-ended (Nakagawa et al., 2013) questions for students with developmental disabilities. Nakagawa et al. (2013) compared the training effects of WSUR training and *segment-unit reading* (SUR) training with one student with ASD. WSUR training required the participant to read whole sentences repeatedly, whereas SUR training required him to read the segments sequentially in their correct spatial locations within sentences. Each word or segment was presented individually during SUR training, which increased the focus on the presented stimuli. Results indicated that one student with ASD improved reading fluency through both types of repeated reading. However, his reading accuracy and comprehension of literal questions only improved after the SUR training. Omori and Yamamoto (2018) investigated SUR training for children with ASD and LDs with intellectual disabilities and reported that training improved reading comprehension of closed-ended questions. The participants could not read and comprehend whole sentences in the baseline phase, whereas they learned to comprehend whole sentences by matching sentences with corresponding pictures after training. These results suggest that repeatedly presenting each segment facilitates comprehension in Japanese students with developmental disabilities as assessed by a matching task (Omori & Yamamoto, 2018) and verbal expressions (Nakagawa et al., 2013). However, previous studies did not directly compare the training effects of the two repeated reading procedures in students with ASD and LDs. In addition, it is yet unknown how typically developing (TD) students would differentially improve their reading comprehension by these two training techniques.

## Objectives

We examined whether 8 students with ASD, 7 students with LDs, and 13 TD students improved their reading accuracy, fluency, and comprehension of open-ended questions through SUR training and WSUR training. Participants were required to read whole sentences and answer five questions about the sentences pre- and posttest under SUR and WSUR training conditions. Shorter texts facilitate the reading comprehension skills of individuals with developmental disabilities (FCRR, 2006; Potocki et al., 2015). Therefore, we predicted that students with reading difficulties would show greater reading comprehension improvements after SUR training than after WSUR training (Nakagawa et al., 2013; Omori & Yamamoto, 2018), whereas TD students would show similar improvements in reading comprehension skills after both types of training.

## Method

### Participants

Participants were students (*N* = 28, 20 boys and 8 girls: 8 with ASD, 7 with LDs, and 13 TD, ranging in age from 9 to 15 years). Participants with ASD and LDs had been diagnosed by a pediatrician using the criteria from the *Diagnostic and Statistical Manual of Mental Disorders*, 5th edition (American Psychiatric Association, 2010). Informed written consent was obtained from the participants’ parents before the study was conducted. We matched the mean chronological ages of the student groups (ASD = 12.38; LD = 11.71; TD = 12.62). All the participants were enrolled in mainstream classes in a public elementary school or junior high school. None of the students with ASD or LDs had any notable social, communication, or behavioral problems other than reading comprehension. Participants were assessed using the Wechsler Intelligence Scale for Children, 4th edition (Japanese edition; Wechsler, 2010). Their mean full-scale IQ (FSIQ) was 99.25 (*SD* = 6.31). TD students had a higher mean FSIQ (104.92, *SD* = 7.40), *F*(2, 25) = 5.23, *MS* = 253.15, *p <* .05, *r* = .42, than students with ASD (96.25, *SD* = 5.42), *t*(19) = 2.77, *p* < .05, and students with LDs (96.57, *SD* = 6.48), *t*(18) = 2.56, *p* < .05.

We assessed the participants’ hiragana (a Japanese syllabary consisting of 46 characters) reading skills using four one-line sentences (16.00 letters, *SD* ± 2.00) that included first- to second-grade-level kanji characters (Japanese ideograms) in a sentence. All the participants could accurately read all the hiragana and kanji characters used in the four short sentences within 3 s.

### Stimulus and Apparatus

A desktop computer (Dell TruStudioPC with Windows 7) was used to control the presentation of pre- and posttest stimulus passages on a display (Iiyama ProLite E2710HDS, resolution 1920 × 1080 pixels). A laptop computer (Panasonic Let’s Note CF-S10 with Windows 7) was also used to conduct the training.

We prepared 50 passages written in hiragana and kanji. Each passage was presented on two slides in 28-point font. A half-width space was inserted between each segment, and one line space was inserted between lines. A passage consisted of 66.25 segments (range 50–85) and a mean of 284.04 letters (range 248–318). Examples of the passages’ topics included “modes of life,” “origins of food,” “origins of sports,” and “science and technology.” Each passage contained five factual questions related to the passage focusing on “what,” “where,” “when,” “who,” “why,” or “how.”

### Procedure

A pre-post design was used to assess the effects of the two training conditions: SUR training and WSUR training.

#### Pretest

Each passage and its associated questions were presented sequentially on the computer. Participants were instructed to read the story aloud on each of the two slides as quickly as possible and verbally answer questions. After the participant read the two slides displaying the passage, five questions were sequentially presented on the computer to the participant. Participants were instructed to answer all the questions, if possible, sequentially. They were required to respond with “I don’t know” if they could not answer a question. Previously, we had matched the passages for reading accuracy, reading time, and reading comprehension after each participant had read 6 to 10 passages and answered questions. The participants began the training using six passages with matching reading levels; 14 participants were trained with SUR training on the first, third, and fifth passages and WSUR training on the second, fourth, and sixth passages, whereas the other 14 participants were trained in the opposite order.

#### Training Conditions

**Fig. 1:**
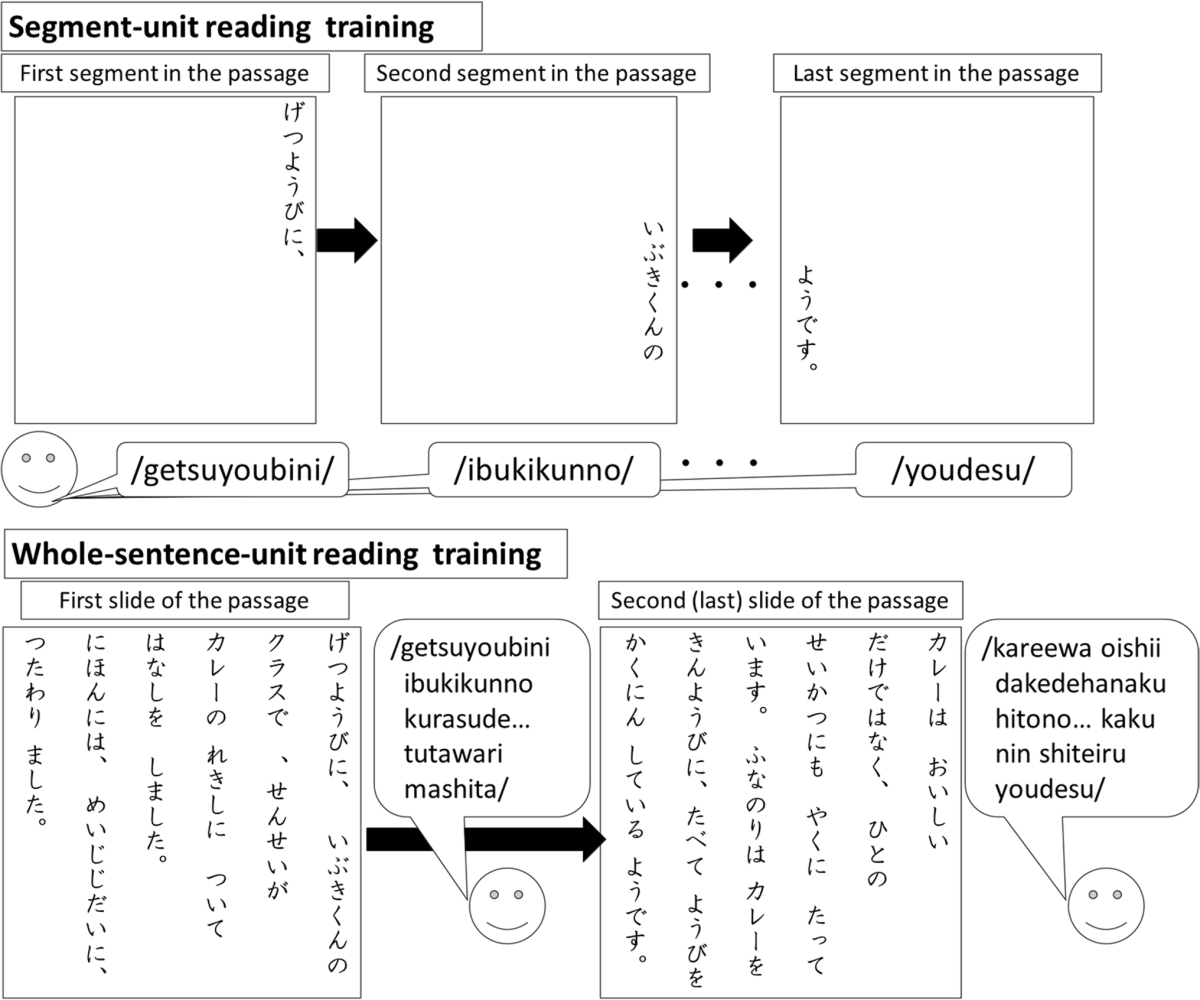
The two types of repeated reading procedures. *Note.* The top panel shows the segment-unit reading procedure (SUR) training. After each presented segment is read in SUR training, the next segment’s location is moved from the top to the bottom. The bottom panel shows the whole-sentence-unit reading procedure (WSUR) training. A segment consisted of one meaningful word and one meaningless particle

Each participant was randomly assigned three passages used in the pretest to SUR and WSUR training conditions. The training order was counterbalanced. Presenting one training condition was followed by the posttest for one of the six passages. Following this, the participants began the other type of training using a different set of three passages. Figure 1 shows the two reading training conditions: SUR (top) and WSUR (bottom).

In SUR training, only one segment of a passage was presented on the computer screen at one time, and participants were required to read the single segment aloud. After the participants had read it accurately, the experimenter clicked on the slide, and the subsequent passage segment appeared. Each segment of pre- and posttest passages appeared in its correct location after the preceding segment disappeared. Therefore, a participant moved their eyes from top to bottom during training because Japanese passages are read vertically. The experimenter did not present the next slide if a participant could not read the segment accurately or correctly, and the participant attempted to reread the segment. If a participant could not read a word or a segment accurately after two presentations, the experimenter pointed with their index finger at the targeted word or segment as a prompt, demonstrated the vocal sound of the word or segment, and instructed the participant to reread the segment. The participant had to read all the passage segments sequentially in each SUR training block, and the participant was given two training blocks.

In WSUR training, the first slide of a passage was presented, and participants were required to read all the presented sentences aloud. After reading the first slide of a passage, the second slide was immediately presented, and participants read the sentences aloud. Similar to prompting in SUR training, the experimenter pointed to the target and demonstrated the spoken sound if a participant could not read a word or segment accurately after two presentations. After the experimenter demonstrated the vocal sound, the participant was instructed to read the segment once. The following slide was presented if the participant could read the segment correctly. The participants read all the passages in one WSUR training block, and each participant responded to two training blocks. The participants started the posttest immediately after the training. After completing one training and posttest session, the participants began training on the remaining passages using the other training method. This training and testing cycle continued until the participants completed training on all six passages.

#### Posttest

The posttests were identical to the pretests. The same stimulus texts were used in pre- and posttests because previous studies (Nakagawa et al., 2013; Omori & Yamamoto, 2018) have demonstrated that students with developmental disabilities often fail to improve their reading comprehension skills even when the same stimulus passages were used in pre- and posttests.

### Dependent Variables

Three dependent variables were used to evaluate reading skills improvements: accurately read segment percentages, reading time, and correct response percentages to questions about the passages. We counted the number of phonetically correct verbal responses to the segments in pre- and posttests compared to the number of vocal responses to calculate reading accuracy. Moreover, we measured reading time from the participant reading the first segment to the participant reading the last segment of a passage to calculate reading time. Furthermore, we counted the number of correct responses to the five questions about each passage and calculated the percentage of correct responses as a measure of reading comprehension.

### Reliability

Two independent observers, including the experimenter, Omori, involved in the testing, evaluated whether a correct response was provided in the reading comprehension test. Both observers listened to the participants and independently evaluated whether the response was correct. The observers evaluated all the trials for each participant. Trial-by-trial interobserver agreement (IOA) was calculated as the number of consistent, correct responses. The IOA values were 100% for pre- and posttest reading comprehension tests. Cohen’s kappa (Cohen, 1968) was calculated to measure interrater reliability for vocal responses, which was 1.00 for all the participants’ reading comprehension test responses.

### Data Analysis

We conducted a mixed-factorial analysis of variance on reading accuracy, fluency, and comprehension scores using a 3 (group: participants with ASD or LDs and TD students; between group) × 2 (training conditions: SUR and WSUR; within group) × 2 (test conditions: pretests and posttests; within group) design to compare the effects of training type on the performance of the two groups of participants.

## Results

### Reading Fluency and Accuracy

Table 1 shows the mean reading time, the mean percentage of reading accuracy, and the mean percentage of correct responses on the comprehension quiz for the three groups of participants.

**Table 1:** Reading skills results of all participants

Diagnoses	Participants	Age	FSIQ	Reading time (s)				Reading Accuracy (%)				Reading Comprehension (%: n/5)			
				WSUR		SUR		WSUR		SUR		WSUR		SUR	
				Pre	Post	Pre	Post	Pre	Post	Pre	Post	Pre	Post	Pre	Post
ASD	ryo	10	94	96.30	92.35	107.20	81.55	85.82%	87.21%	81.88%	91.44%	30.00%	40.00%	20.00%	70.00%
	daiki	11	103	67.80	83.51	57.85	77.41	82.52%	87.98%	86.79%	95.30%	10.00%	30.00%	30.00%	70.00%
	kouki	11	103	66.48	66.07	128.23	60.71	87.76%	89.27%	87.94%	88.00%	20.00%	50.00%	20.00%	90.00%
	shota	11	90	123.55	74.47	151.40	95.18	81.21%	92.10%	78.07%	90.51%	20.00%	30.00%	20.00%	80.00%
	kei	13	99	73.78	47.15	119.34	54.05	93.28%	93.86%	90.02%	97.87%	40.00%	50.00%	40.00%	90.00%
	ken	13	99	106.32	91.42	94.99	99.09	89.64%	93.28%	85.51%	96.10%	20.00%	20.00%	10.00%	90.00%
	ren	15	91	53.08	60.59	62.32	74.02	91.04%	83.42%	90.70%	88.10%	30.00%	40.00%	20.00%	70.00%
	rui	15	91	123.96	84.04	118.44	97.91	79.83%	93.50%	87.56%	95.50%	40.00%	50.00%	20.00%	90.00%
	saki	8	87	150.50	67.84	125.26	66.00	89.22%	92.26%	88.11%	95.47%	20.00%	50.00%	30.00%	90.00%
	haruto	11	105	103.00	74.09	105.55	106.54	78.58%	94.15%	86.70%	84.29%	50.00%	80.00%	50.00%	90.00%
	aya	11	93	72.70	53.85	80.86	60.82	91.10%	96.02%	88.98%	94.65%	20.00%	30.00%	30.00%	90.00%
LD	atsuto	11	105	126.70	96.38	91.99	75.18	86.96%	93.11%	91.96%	97.10%	0.00%	80.00%	0.00%	40.00%
	shun	13	98	81.87	89.62	97.37	68.78	87.27%	86.64%	87.54%	94.42%	30.00%	70.00%	20.00%	60.00%
	kai	13	98	76.68	61.28	101.43	89.50	84.30%	97.12%	85.03%	97.66%	30.00%	60.00%	10.00%	80.00%
	hide	15	90	83.98	73.74	85.77	56.38	87.65%	98.08%	84.22%	98.36%	30.00%	50.00%	30.00%	80.00%
	jo	9	105	85.17	88.08	67.35	83.78	90.18%	96.38%	88.02%	98.24%	40.00%	80.00%	30.00%	100.00%
	yoko	11	110	65.64	71.01	107.64	67.81	89.86%	97.10%	88.48%	96.67%	30.00%	60.00%	20.00%	100.00%
	mei	11	99	106.50	44.26	120.76	54.89	80.18%	95.66%	83.74%	93.56%	30.00%	70.00%	10.00%	60.00%
	jun	11	108	101.60	49.11	77.95	90.79	93.61%	96.53%	90.50%	90.49%	40.00%	80.00%	30.00%	80.00%
	sana	12	121	70.82	106.21	69.14	110.43	91.00%	99.00%	91.50%	94.50%	40.00%	60.00%	30.00%	80.00%
	rin	12	94	70.09	75.92	69.57	54.97	81.00%	95.18%	84.51%	95.94%	30.00%	60.00%	40.00%	80.00%
TD	masaki	12	115	88.26	85.81	115.49	78.17	90.12%	95.65%	95.10%	94.65%	20.00%	80.00%	30.00%	70.00%
	taku	13	110	58.12	42.47	59.00	43.65	94.03%	98.38%	89.69%	98.50%	30.00%	100.00%	40.00%	80.00%
	rei	13	98	95.79	117.79	114.78	111.36	91.35%	88.73%	89.64%	89.82%	30.00%	80.00%	30.00%	90.00%
	gen	15	101	150.26	94.46	84.79	98.02	92.43%	96.51%	92.50%	97.00%	20.00%	70.00%	20.00%	70.00%
	kota	15	100	87.80	74.85	100.67	60.35	85.55%	92.50%	86.67%	91.75%	10.00%	70.00%	30.00%	60.00%
	tomo	15	105	64.10	50.40	64.10	59.05	90.00%	98.20%	90.00%	97.50%	40.00%	90.00%	40.00%	90.00%
	mie	15	98	137.97	77.14	132.61	118.66	89.12%	97.82%	92.10%	100.00%	10.00%	80.00%	20.00%	80.00%
Mean	ASD	12.38 (0.68)	96.25 (1.92)	88.91 (9.68)	74.95 (5.65)	104.97 (11.36)	79.99 (5.99)	**86.39%** (0.02)	**90.08%** (0.01)	**86.06%** (0.01)	**92.85%** * (0.01)	26.25% (0.04)	**38.75%** (0.04)	22.50% (0.03)	**81.25%** ** (0.04)
	LD	11.71	96.57	99.35	73.83	98.32	74.74	86.44%	93.91%	87.51%	94.56%	25.71%	**60.00%**	24.29%	**75.71%** **
		(0.84)	(2.64)	11.07)	(5.67)	(5.54)	(6.68)	(0.02)	(0.01)	(0.01)	(0.02)	(0.06)	(0.07)	(0.06)	(0.07)
	TD	12.62 (0.54)	104.92 (2.13)	90.93 (7.78)	75.19 (6.59)	91.07 (6.98)	79.38 (6.89)	**89.11%** (0.01)	**95.97%** (0.01)	**89.42%** (0.01)	**95.28%** (0.01)	28.46% (0.03)	**75.38%** (0.03)	28.46% (0.02)	80.00% (0.04)
	ALL	12.32 (0.37)	100.36 (1.51)	92.46 (5.18)	74.78 (3.62)	96.85 (4.76)	78.39 (3.89)	87.66% (0.01)	93.77% (0.01)	87.98% (0.01)	94.41% (0.01)	27.14% (0.02)	61.07% (0.04)	25.71% (0.02)	79.29% (0.03)

*Note.* Standard error scores are indicated underneath each mean score. Reading comprehension scores are the results of answering open-ended literal/factual questions. The underlined scores indicate there was no increase or decrease after training. The scores in bold indicate the results of multiple comparison tests showing greater reading accuracy in typically developing (TD) students than students with autism spectrum disorder (ASD). WSUR = whole-sentence-unit reading, SUR = segment-unit reading; LD = learning disability; FSIQ = full-scale IQ

* *p* < .05. ** *p* < .01

All the students spent an average of 92.46 s reading time (*SE* = 5.18) for WSUR pretests and 96.85 s reading time (*SE* = 4.76) for SUR pretests. They decreased their reading time to 74.78 s (*SE* = 3.62) after WSUR training and 78.39 s (*SE* = 3.89) after SUR training. There was a significant main effect of the test conditions, *F*(1, 25) = 9.11, *MS =* 6201.77, *p* < .01, *r =* .52, which indicated that SUR training and WSUR training were effective in decreasing reading time in all the study groups.

All the students demonstrated an average 87.66% reading accuracy (*SE* = 0.01) in the WSUR pretest and 87.98% reading accuracy (*SE* = 0.01) for the SUR pretest. They increased their reading accuracy to 93.77% (*SE* = 0.01) after WSUR training and 94.41% (*SE* = 0.01) after SUR training. There were main effects of group, *F*(2, 25) = 4.63, *MS =* .011, *p* < .05, *r* = .40, and test condition, *F*(1, 25) = 53.33, *MS =* .010, *p* < .001, *r =* .83. A multiple comparison using Ryan’s method revealed that students with ASD (88.84%) had lower overall reading accuracy than TD students (92.44%), *t*(82) = 3.25, *MSE* = .002, *p* < .005, *r =* .34. All the students successfully improved their reading accuracy after both types of training, such that the participants decreased their reading errors from 8.41 times of errors (*SE* = 1.14) to 4.43 times (*SE* = 0.81) after WSUR training and 8.17 times of errors (*SE* = 0.90) to 3.82 times (*SE* = 0.92) after SUR training.

### Reading Comprehension

The students scored an average 27.14% (*SE* = 0.02) and 25.71% (*SE* = 0.02) for reading comprehension in WSUR and SUR training pretests, respectively. They improved their reading comprehension to 61.07% (*SE* = 0.04) after WSUR training and 79.29% (*SE* = 0.03) after SUR training. We found a significant group, training, and test condition interaction, *F*(2, 25) = 6.87, *MS* = .099, *p* < .005, *r =* .47. A post hoc analysis using three simple-simple main effects tests revealed a simple-simple main effect of group and posttest for WSUR training, *F*(2, 100) = 18.30, *MS* = .029, *p* < .001, *r =* .39, whereas there was no simple-simple main effect of SUR training, *F*(2, 100) = .46, *MS* = .007, *p* = .64, *ns*. A multiple comparison using Ryan’s method revealed that students with ASD showed less reading comprehension improvements after the WSUR training (38.75%) compared to those with LDs (60.00%), *t*(13) = 3.24, *MSE* = .016, *p* < .005, *r =* .60, and TD students (75.38%), *t*(19) = 6.43, *MSE* = .016, *p* < .001, *r =* .83, whereas TD students showed better reading comprehension improvements than students with LDs, *t*(18) = 2.59, *MSE* = .016, *p* < .05, *r =* .52.

We also found simple-simple main effects of training in participants with ASD at posttest, *F*(1, 50) = 54.89, *MS* = .786, *p* < .001, *r* = .72, and participants with LDs’ posttest percentages, *F*(1, 50) = 7.50, *MS* = .107, *p* < .01, *r* = .36, indicating that students with ASD (81.25%) and students with LDs (75.71%) had a higher percentage of correct responses after SUR training than after WSUR training (38.75% and 60.00%, respectively). To the contrary, TD students improved their reading comprehension equally after both types of training (WSUR: 75.38%; SUR: 80.00%), *F*(1, 50) = .68, *MS* = .009, *p* = .435, *ns*. These results indicated that ASD students found it more difficult to comprehend a passage after WSUR training than students with LDs and TD students, similar to students with LDs compared to TD students. Students with ASD and LDs showed higher percentages of correct responses after SUR training than after WSUR training, whereas TD students showed similar results after both types of training.

## Discussion

### Improved Reading Comprehension Skills with WSUR and SUR Training

This study examined whether students with ASD, students with LDs, and TD students improve their reading skills through two types of repeated reading training: SUR and WSUR. Table 1 shows that most students improved their reading accuracy, reading time, and comprehension after training, and both types of training had comparable effects on improving reading accuracy and decreasing the reading time of all the study groups. Moreover, TD students improved their reading comprehension equally after both training conditions. However, the results indicated that students with ASD and students with LDs scored higher on the comprehension quiz after the SUR training than after the WSUR training, which replicated Omori and Yamamoto (2018) and Nakagawa et al. (2013). Students with ASD and LDs were more likely to improve their reading comprehension skills assessed by open-ended literal questions when each segment of a passage appeared discretely within a temporal and spatial sequence.

### Effects of Sequentially Presenting Short Stimuli

Participants read whole sentences repeatedly in typical repeated reading training (Ambruster et al., 2003). However, research has indicated that students with ASD and LDs can read and comprehend words (Faggella-Luby & Deshler, 2008; Frith, 1986; Nation et al., 2006). Previous research has shown that presenting words (FCRR, 2006) or short texts (Potocki et al., 2015) assisted students with developmental disabilities in decreasing their reading time and increasing their comprehension of closed-ended questions. This study indicates that although English and Japanese have different writing systems, sequentially presenting Japanese segments assisted students with developmental disabilities in answering open-ended questions. There are usually no spaces between words or letters in Japanese sentences, and finding segments in sentences can be a key to comprehending a passage and its context. We presented spaced sentences in pre- and posttests; however, sequentially presenting each segment separately during training might have made it easier for participants to read and comprehend the meaning of these segments. Conversely, presenting whole sentences of a passage on the display might have decreased these participants’ chances of comprehending the meaning of each segment, which might explain why participants with developmental disabilities showed more significant improvements in reading comprehension after SUR training than after WSUR training.

### Why Is Presenting the Correct Location Necessary for Students with Developmental Disabilities?

Developing the ability to read a passage fluently, by itself, might not directly result in the comprehension of the passage (Stevens et al., 2017). In SUR training, we presented each passage segment sequentially in its correct location, which was expected to help participants observe the letter strings as words, visually identify the words, and integrate the passage’s meaning (Omori & Yamamoto, 2018). In other words, controlling the quantity and the location of stimuli was expected to facilitate reading comprehension of passages by individuals with developmental disabilities. Moreover, students with ASD and students can relate tools and component skills to passage reading comprehension as composite behaviors (Johnson & Layng, 1992, 1996) after becoming capable of reading and comprehending segments (FCRR, 2006).

### Japanese Students with Developmental Disabilities’ Difficulties in Reading Whole Sentences

Table 1 shows that students with ASD had the most negligible reading comprehension improvements, and students with LDs had intermediate improvements after WSUR training. Previous studies have shown that Japanese students with developmental disabilities often struggle with identifying meaningful words from sentences without spaces (Kuhara-Kojima et al., 1996; Takahashi et al., 2011). Even though we used spaced sentences, Japanese students with developmental disabilities had difficulties dividing components of simultaneously presented sentences and connecting meanings. WSUR training with Japanese sentences also facilitated reading speed and accuracy improvements in students with developmental disabilities, possibly due to characteristics of the Japanese language. Japanese hiragana letters have a point-to-point correspondence between letters and sounds, and reading itself is not very difficult for Japanese students with developmental disabilities. However, increasing reading fluency might not be sufficient for Japanese students with developmental disabilities to improve sentence reading comprehension. Why was WSUR training not as effective as SUR training for Japanese students with developmental disabilities, and why did TD students improve their reading comprehension skills by repeatedly reading whole sentences? These issues remain to be investigated.

### Future Research and Limitations

Omori and Yamamoto (2018) reported the results of only two participants. In contrast, the current study extended the applicability of SUR training to improving the reading comprehension skills of students with developmental disabilities and reading difficulties. Previous studies have shown that it is difficult to improve the reading comprehension of students with intellectual disabilities by having them repeatedly read long sentences (Nakagawa et al., 2013; Omori & Yamamoto, 2018). Participants with ASD and LDs in this study had not been diagnosed with intellectual disabilities, and their FSIQs ranged from 87 to 105. Nevertheless, the small sample of participants with developmental disabilities showed greater reading comprehension improvements after SUR training. Moreover, two students with LDs showed more significant comprehension improvements after WSUR training, similar to previous studies (Ambruster et al., 2003; Fuchs et al., 2001; Stevens et al., 2017), whereas all the students with ASD only showed a slight improvement. Therefore, further research is required to identify whether training effects on developmental disabilities differ based on intelligence, diagnoses, or an interaction between them. It is suggested that future studies investigate the responsiveness of students with developmental disabilities to SUR training interventions by analyzing their behavioral and cognitive profiles precisely.

The training time might be another potential confounding factor in this study. We did not record the reading time during training, and the participants might have required a shorter training time to complete WSUR training than SUR training. Future research must analyze the behavioral repertoires of participants with developmental disabilities to evaluate their responsiveness to SUR training. There might also have been possible testing effects caused by the pre-post design of this study, and a no-training group or a waiting-list group of participants should be included to examine testing effects in future studies. Preparing untrained sentences could also be a solution for canceling the testing effects of SUR training, as indicated in a previous study (Omori & Yamamoto, 2018). One reason for the difficulties faced by Japanese students with developmental disabilities in reading whole sentences could be the difference in eye-movement patterns during reading (Omori, 2019). Omori (2019) reported that TD students read whole sentences with their eyes focused on segments, whereas students with developmental disabilities mainly focused on each letter in a sentence. Therefore, future research should analyze eye-movement changes along with reading comprehension development to clarify why SUR training and WSUR training have different effects, especially on students with developmental disabilities.

## Data Availability

Not applicable.
